# The *All of Us Evenings with Genetics* Research Program

**DOI:** 10.1017/cts.2026.10763

**Published:** 2026-06-16

**Authors:** Stacy M. Lloyd, Jasmine Baker, Carolina Jorgez, Julie Coleman, LaTerrica Williams, April Adams, Ashley Butler, Elizabeth Atkinson, Shamika Ketkar, Steven Scherer, Kim Worley, Susan Fernbach, Keasia Daniels, Latanya Hammonds-Odie, Laura Rosales, Brendan Lee, Debra Murray

**Affiliations:** 1 Pathobiology, School of Veterinary Medicine, Tuskegee University, Tuskegee, AL, USA; 2 Pediatrics, Baylor College of Medicine, Houston, TX, USA; 3 Urology, Baylor College of Medicine, Houston, TX, USA; 4 Molecular and Human Genetics, Baylor College of Medicine, Houston, TX, USA; 5 Pediatrics-Psychology, Baylor College of Medicine, Houston, TX, USA; 6 Obstetrics and Gynecology, Baylor College of Medicine, Houston, TX, USA; 7 VantagePoint Business Solutions, Houston, TX, USA; 8 Biological Sciences, Georgia Gwinnett College, Lawrenceville, GA, USA

**Keywords:** *All of Us* database, *All of Us* Researcher Workbench, precision medicine, biomedical research, early career scientist

## Abstract

The *All of Us Evenings with Genetics* (*All of Us* EwG) Research Program, funded in part by the National Institute of Health’s All of Us Research Program, engages researchers to utilize the All of Us Researcher Workbench to advance precision medicine. The All of Us EwG program has two core aims: (1) address barriers to research productivity for minorities, disadvantaged, and persons with disabilities aspiring to enter STEM fields; and (2) promote the use of the All of Us Researcher Workbench to develop research focused on health equity. To achieve these goals, the program implements three key activities: (1) Campus Seminars: hosted at minority-serving institutions and smaller universities to connect faculty with the All of Us Research Program to showcase ongoing research; (2) Biomedical Researchers Faculty Summit: a multi-day conference for early career researchers to foster collaboration and train participants in using the All of Us database; and (3) Biomedical Researcher Scholars Program: A yearlong initiative supporting the research teams formed at the summit in implementing the research projects developed using the All of Us data. Overall, the All of Us EwG program offers data science training, mentorship, and professional development; this report outlines its structure, outcomes, and lessons learned.

## Introduction

Scientific discoveries have significantly improved the overall health of the U.S. population; however, racial/ethnic minorities, socioeconomically disadvantaged, and under-resourced rural populations continue to experience a disproportionate burden of disease and adverse health outcomes [1]. Ironically, underrepresented (UR) groups with higher burdens of chronic medical conditions, such as asthma, certain cancers, diabetes, heart disease, and stroke, may not experience any benefit from “personalized medicine,” due to the lack of representation in genomic databases. This lack of representation constrains research evaluating genomic associations with chronic disease, limiting accurate risk prediction and personalized care for non-European and minoritized populations [2–4]. Simultaneously, scientists from UR communities, who often work in institutions with limited research infrastructure, encounter barriers to accessing large health databases, including limited awareness, insufficient training in genomic data science, and restricted access to computational resources, further limiting the generation of evidence relevant to UR populations [5]. To address this lack of representation, the *All of Us* Research Program endeavored to accelerate health research and medical breakthroughs to enable individualized prevention, treatment, and care by delivering one of the largest, richest biomedical datasets available to registered institutions to support the additional research needed to inform strategies to ameliorate disparities. The *All of Us* database provides an opportunity to understand the various factors that contribute to health for all Americans and was developed as part of the Precision Medicine Initiative, launched by the Obama administration [6,7].

With a concerted effort to include UR groups, *All of Us* recruited a nationwide research cohort of approximately one million volunteers to propel our understanding of health and disease and set the foundation for a new way of doing research through engaged participants and open, responsible data sharing [8]. The *All of Us* database can be accessed and analyzed by registered researchers on the *All of Us* Researcher Workbench (https://workbench.researchallofus.org). The *All of Us* Researcher Workbench is a cloud-based computational platform that utilizes standard programming languages, such as R and Python, and applications, such as Jupyter Notebook, SAS Studio, and RStudio. The data include electronic health records, Fitbit data, physical measurements, survey responses (e.g., lifestyle, environmental exposures, social determinants of health), genotyping arrays, and whole genome sequences. The *All of Us* program emphasized the enrollment of racial (defined as those who self-identify as American Indian or Alaska Native, Asian, Black or African American, Native Hawaiian or Other Pacific Islander, and White), and ethnic (Hispanic or Latino; Not Hispanic or Latino) minority populations [9], while also designating nine other groups as historically UR (individuals with limited access to care, low income, low educational attainment, cognitive or physical disabilities, residing in rural areas, under 18 or over 65 years of age, intersex and sexual or gender minorities) (Supplemental Figure 1). As of August 2025, *All of Us* has successfully enrolled more than 867,000 individuals, of whom >590,496 have completed the initial steps of the program; >45% were from racial and ethnic minority groups and >75% were from communities UR in biomedical research [10,11].

Additionally, over 18,000 researchers from 1161 organizations worldwide had registered on the Researcher Workbench. Of 859 academic institutions with an executed data user agreement (DURA), 132 (15%) were Historically Black Colleges and Universities (HBCUs) and Hispanic-Serving Institutions (HSIs). Among registered users, 78% were UR in biomedical research, and 21% were racial/ethnic minorities. To estimate usage of the *All of Us* Researcher Workbench, we quantified active users by the development of “workspaces” within the Researcher Workbench, i.e., project spaces on the Researcher Workbench where projects are stored and analyzed. Approximately 50% (8400) of researchers were active users, including 38% (6500) UR and 5% (900) racial/ethnic minority researchers. The figures for UR and racial/ethnic minorities actively using the database are significantly less than those mentioned earlier for all registered users [12]. To support broader researcher engagement with the *All of Us* data, the *All of Us Evenings with Genetics* (*All of Us* EwG) Research Program was born.

Developed at Baylor College of Medicine (BCM), the *All of Us* EwG Research Program aims to increase the number of UR biomedical science researchers using the Researcher Workbench, while advancing projects that address chronic diseases and health disparities. To accomplish these goals, we undertook three major activities: (1) *All of Us* EwG Campus Seminars, (2) *All of Us* EwG Biomedical Researchers Faculty Summit (BRFS), and (3) a twelve-month *All of Us* BR Scholars Program (BRSP). These activities engaged UR researchers across career stages, disciplines, and academic institution types, including Minority-Serving Institutions (MSIs) and both teaching and research-intensive institutions. Each initiative aimed to raise awareness and promote the use of the Researcher Workbench as a tool to confront persistent health disparities, led by researchers who understand the social and structural contexts of these issues.

## *All of Us* EwG campus seminar

A large portion of U.S. colleges and universities had a DURA agreement, including a notable representation from HBCUs and HSIs. Generally speaking, MSIs (i.e.), HBCUs, HSIs, Tribal Colleges and Universities, Alaska Native and Native Hawaiian Serving Institutions (ANNH), Asian American and Native American Pacific Islander Serving Institutions (AANAPISIs), Predominantly Black Institutions (PBIs), and Native American Serving Nontribal Institutions (NASNTIs) largely attract students from communities of color and those in the bottom two income quintiles [13]. The vast majority of MSIs are heavily supported by public funding, due to the inability to rely on student tuition as a means of revenue. For example, nearly 70% of all revenue for public two-year HSIs comes from state and local sources, relative to 51% for public two-year non-MSIs. Furthermore, 54% of all revenue from public four-year HBCUs comes from federal, state, and local appropriations, grants, and contracts, compared to 38% of public non-HBCU four-year institutions [13]. Interestingly, enrollment-based MSIs that attain designation by meeting enrollment thresholds for specific minority student populations (e.g., HSIs, PBIs, ANNH, AANAPISIs, and NASNTIs) must maintain low educational and general expenditures to retain their federal designation and remain eligible for the U.S. Department of Education’s MSI capacity building grant programs. This requirement, in turn, perpetuates resource limitations, particularly in research and technological infrastructure [14]. In contrast, HBCUs, which are mission-oriented, have slightly different funding structures. A significant portion of revenue for HBCUs comes in the form of federal funding, state and local appropriations, grants, and contracts. In 2015, public four-year HBCUs received an estimated $2.2 billion in federal, state, and local appropriations, while public four-year non-HBCUs received a staggering $94 billion from the same sources [13,14]. Beginning in 2021, the Biden-Harris administration announced over $16 billion in federal investments to HBCUs during Fiscal Years (FY) 2021 through 2024, encompassing grants, debt relief, student aid, as well as research and infrastructure assistance. Despite recent support, funding for HBCUs remains insufficient to sustain research capacity and infrastructure development [15].

As a means of increasing the utilization of the *All of Us* Researcher Workbench by researchers and scientists who are UR in the biomedical sciences, the *All of Us* EwG Campus Seminar focused on raising awareness of the database at MSIs, to reach diverse researchers (faculty, staff, and students) and their alumni while simultaneously recruiting early career faculty members and senior postdoctoral trainees to the BRFS (discussed below). Modeled after BCM’s Molecular and Human Genetics Department’s highly successful *Evenings with Genetics* (EwG) program, which brought together experts (clinicians, researchers, patient advocates, and caregivers) with families impacted by genetic conditions, to provide education, resources, and support using plain language. The *All of Us* EwG Campus Seminar was facilitated by an *All of Us* EwG team member and included research presentations from four campus faculty members and their students. Topics addressed included the currently available *All of Us* data, student-appropriate hypotheses tailored to learners’ interests and their current level of knowledge, and the potential for long-term engagement. We also highlighted ways faculty members from non-research-intensive institutions could develop *All of Us* research projects that could be integrated into their curriculum.

## *All of Us* EwG BRFS

Early career researchers (defined as research associates, faculty, and/or scientists) [16], and senior postdoctoral trainees, were recruited from across the United States by emailing institutions (behavioral, education, social science, and STEM department chairs and faculty members), early career faculty groups (e.g., American Society of Human Genetics’ Human Genetics Scholars Initiative), postdoctoral organizations (National Postdoctoral Association, National Black Postdocs), professional science societies (American Society for Cell Biology, National Science Foundation HSI STEM Hub, American Association of Cancer Research), posting on social media (Facebook, LinkedIn, Twitter), and promoting at research conferences (e.g., Annual Biomedical Research Conference for Minoritized Scientists) to compete for participation in the BRFS. U.S. and non-U.S. citizen applicants who identified as early career faculty (Instructor, Assistant Professor) or senior postdoctoral trainee (a year from seeking an academic position), expressed an interest in the *All of Us* database, and were currently working at a U.S. academic institution were considered for admission.

The four-day BRFS training schedule emphasized the necessity of networking and collaboration through the development of multidisciplinary research teams and the identification of a team project to be completed on the *All of Us* Researcher Workbench. Scholars were not denied admission to the BRFS if their institution did not have an executed DURA at the time of application. If scholars were accepted into the program, with the support of the *All of Us* EwG program staff, they worked with their institution to apply for a DURA. Providing structured support for this process helped mitigate administrative and institutional barriers that can disproportionately limit participation by scholars from under-resourced institutions.

Because the *All of Us* Researcher Workbench is a cloud-based computational platform that utilizes standard programming languages, such as R and Python, and applications such as Jupyter Notebook, SAS Studio, and RStudio, scholars received four “data science” training sessions to gain basic skills in accessing the Researcher Workbench and the numerous data types available: demographics, EHR, Fitbit data, physical measurements, survey responses, and genomic data. Through step-by-step instructions and demonstrations, the training provided detailed information to successfully query and analyze the available data. Coleman et al. and Baker et al. have previously published the curriculum design and development methodologies for these training sessions [17,18].

To provide a supportive and encouraging environment, the scholars were supported by three distinct groups: “The Elders,” team mentors, and data science coaches. “The Elders” represented emeritus-level faculty to provide professional and career coaching. Current BCM assistant, associate, or full professors were recruited to serve as research mentors for each research team formed during the BRFS. The mentors were not required to have expertise in the team’s research topic, but rather to provide guidance in project development, scope of work, and the strategy to accomplish team goals. Importantly, all mentors received training to support the implementation of validated mentorship education interventions, with many completing up to 10 hours of structured instruction. The data science coaches were postdoctoral trainees and graduate students recruited from BCM departments with computational programs (Molecular and Human Genetics, Quantitative and Computational Biosciences) and from laboratories with bioinformatics research projects. Equipped with prior computational expertise, coaches were trained in accessing and analyzing the *All of Us* database before the BRFS began. The coaches then provided one-on-one support to the research teams during the data science training sessions and the evening tutoring sessions.

## *All of Us* EwG BRSP

The twelve-month BRSP began immediately after the BRFS and is designed to provide a sustained learning environment to support the development and implementation of the research projects planned and initiated at the BRFS. Additional data science training sessions on R, Python, and SAS were provided alongside sessions for professional development and ongoing mentoring. Activities to support the teams’ success and research project progression included the development of a management plan to guide their daily operations, participation in an annotated bibliography contest, and application for seed award funding to financially support their work on the Researcher Workbench, in the laboratory, or to support community engagement activities, or disseminate their findings through presentations at national or international conferences. Scholars were encouraged to attend monthly BRSP virtual meetings, to present their research progress. Six months into the program, *All of Us* EwG hosted a One-Day Retreat, designed to engage the scholars for a second face-to-face meeting. This retreat offered additional training on mentoring, data science, grant writing, and the opportunity for teams to meet collectively in a focused setting to maintain a cohesive working environment.

## Program evaluation overview

To assess the effectiveness, efficiency, and impact of the *All of Us* EwG Research Program, we systematically evaluated specific learning activities along with scholars’ perceived usefulness. As part of this evaluation, we surveyed attendees at seminars and campus presentations and documented attendance. Each activity was designed to support scholars’ research interests, career advancement, and provide positive outcome expectations. Quantitative and qualitative assessments were conducted on the following components: (1) *All of Us* EwG BRFS; (2) monthly meetings of the *All of Us* EwG BRSP; and (3) One-Day Retreat. All primary data sources were collected online through a project-specific database developed by the Research Resources Office at BCM.

Upon program acceptance, the scholars completed a background survey about their experience using programming languages and technical tools as well as working with teams to develop and execute research projects. Participants also completed a survey assessing research self-efficacy and self-reported demographic characteristics, including highest degree earned, field of study, and race/ethnicity. During the BRFS and One-Day Retreat, through post-event surveys, the scholars ranked each session by: (1) how well it met its stated objectives, (2) whether new information was presented, and (3) whether it included information applicable to their research. At the conclusion of the BRSP, the evaluation team conducted a structured qualitative interview to assess how participation in the BRSP has benefited each scholar, the quality of their interactions with peers, and other relevant feedback.

## Program outcomes

### All of Us EwG campus seminars

During the funding duration of the *All of Us* EwG Research Program, we collaborated with faculty and staff from four federally designated, Carnegie-classified MSIs: Hampton University (HU), Prairie View A&M University (PVAMU), the University of Texas-El Paso (UTEP), and the University of Texas-Rio Grande Valley (UT-RGV). These doctoral-granting institutions are classified by the Carnegie Classification of Institutions of Higher Education as having either high research activity (R2; annual research expenditures of at least $5 million and conferral of at least 20 doctoral degrees) or very high research activity (R1; annual research expenditures of at least $50 million and conferral of at least 70 doctoral degrees). This designation underscores the collaborative strength of the partnership, which integrates MSIs with shared commitments to equity, complementary research expertise, and access to diverse student populations, thereby enhancing collective training and research capacity [19].

#### UT-RGV (R2)

Part of the University of Texas system, UT-RGV is the 2nd largest HSI in the country, with over 31,500 students [20]. Dr. Bassent Abdelbury was our campus liaison. Dr. Debra Murray presented to the Faculty Research and Professional Development Program, leading to a seminar on March 10, 2022, with 95 attendees. Research presentations covered developing biomaterial for tissue engineering and treatment of cancer, diabetes as a re-emerging contributor to tuberculosis, and autism in Latino children. The seminar led to a new student research experience and the selection of two faculty members for the BRSP.

#### PVAMU (R2)

Texas’s largest and second-oldest HBCU, PVAMU, has an enrollment of 9893 students [21,22]. Dr. Victoria Mgbemena was our campus liaison for the April 7, 2022, seminar with 68 attendees. Research presentations covered DNA-editing enzymes, mental health among Black college students, and cancer cell development via PALB2. The seminar led to new research collaborations, student involvement in a BCM summer program, and recruitment of two faculty members to the BRSP.

#### HU (R2)

Located in Virginia, Hampton University is a private HBCU with 4244 students [23]. Dr. Ayo Akinremi was our campus liaison for the April 22, 2024, seminar with 14 attendees. Research presentations covered gender and leadership, prostate cancer in African American men, and early cancer markers. The seminar led to the creation of the “Hampton Research Hub” focused on COVID-19 and lung infection, which received external seed funding from our program. Two faculty from the Hub were later selected for the BRSP.

#### UTEP (R1)

Also, a part of the University of Texas system, UTEP is the fourth largest HSI nationally with 23,861 students [20] Drs. Renato Aguilera and German Acosta served as campus liaisons for the April 26, 2024, seminar with 35 attendees. Research presentations covered vaccine hesitancy in Hispanic communities, socioeconomic factors in COVID-19 infection, host-pathogen interactions in tuberculosis, and viral sumoylation. Additionally, two former scholars, Drs. Sangeeta Tawari and German Acosta presented BRSP developed projects, sharing their progress and future plans.

### All of Us *EwG BRFS*

From 2022 to 2025, four cohorts of scholars participated in the *All of Us Evenings with Genetics* (EwG) BRFS program. During this period, eligible participants from Campus Seminar schools comprised approximately 5% to 21% of the applicant pool annually. The total number of applicants by year was 41 in 2022, 60 in 2023, 66 in 2024, and 57 in 2025. Applications were reviewed and scored by program staff to determine programmatic fit. Some applications were deemed ineligible and not advanced for review if applicants did not meet minimum eligibility criteria, such as being at an early career stage following completion of a PhD or equivalent degree, with limited exceptions.

In total, 147 scholars (34 in 2022, 34 in 2023, 43 in 2024, and 36 in 2025) were admitted and fully participated in the BRSP by attending the BRFS and providing subsequent survey data. Scholar demographics for the first three cohorts combined were previously published by Baker et al. (2025) 17. Scholar demographics for all four cohorts are stratified by year in Supplemental Figure 2. The scholars represented institutions across a spectrum of research intensity, with 78.8% originating from R1 and R2 institutions as defined by the Carnegie Classification of Institutions of Higher Education (Supplemental Figure 3). Collectively, these data demonstrate the program’s success in recruiting early career UR researchers and introducing them to the *All of Us* Researcher Workbench, while fostering inclusive training and cross-institutional exchange of expertise that strengthened collaboration and amplified the program’s overall impact.

Held annually over four days in May each year in the Texas Medical Center, the BRFS featured scientific plenaries, grant writing, team building, sustainability planning, and data science training. Scholars also received career coaching and training from “The Elders” during roundtable discussions at breakfast and dinner. Each plenary session introduced scholars to experts in the field of genetics who presented their research, shared their scientific career trajectories, and highlighted key experiences that contributed to their professional advancement. In later years, this component was expanded to include alumni scholar teams, providing current scholars with peer-led perspectives and encouragement in developing collaborative research teams and building viable projects on the Researcher Workbench. Team formation began with the “True Colors” personality assessment, grouping scholars by personality type to explore work styles and challenges [24]. Scholars then engaged in “speed dating” based on research interests, organized into 4–5 broad topic areas. Through iterative discussions, teams formed around refined questions and developed management plans outlining roles, communication strategies, meeting schedules, and accountability measures. Each team designated a liaison to coordinate with *All of Us* EwG staff. Data science training, primarily hosted at BCM, taught scholars to set up workspaces, define cohorts, and analyze data in the workspace. Scholars were encouraged to contribute regardless of computer programming experience and textbook resources were provided to assist those with less skill [17]. During the BRFS, scholars evaluated three core components: (1) scientific plenaries; (2) research team development; and (3) data science training. Plenary sessions were rated on a 5-point Likert scale for learning and usefulness. Team development and data science sessions, held over multiple days, were rated individually on a 1–10 scale. Scores were averaged annually, and responses for team and data science sessions were grouped into five categories to align with a 5-point Likert scale.

**Figure 1.**
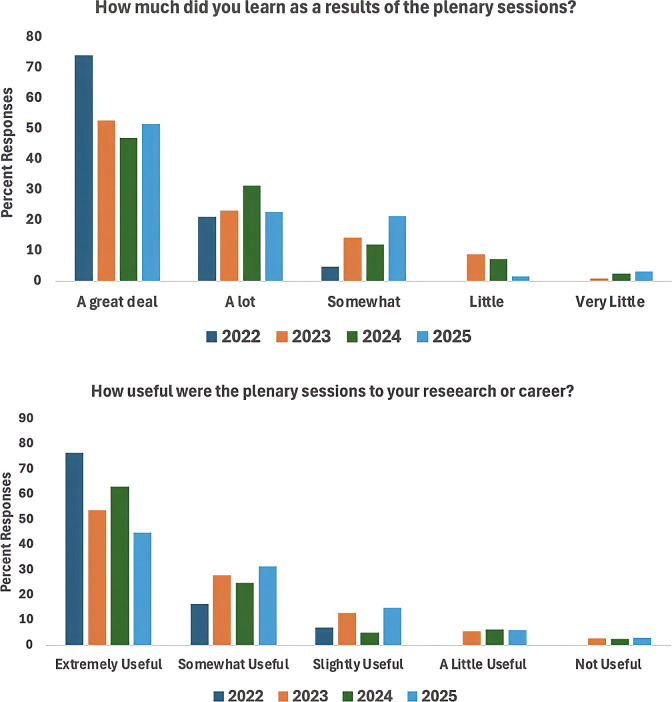
Annual evaluation results from the *All of Us Evenings with Genetics Biomedical Researchers,* Faculty Summit Plenary Sessions. There were three plenary sessions in 2022, four in 2023, two in 2024, and two in 2025. Response counts across sessions and questions varied from *n* = 24 to *n* = 33 in in 2022, *n* = 25 to *n* = 32 in 2023, *n* = 41 to *n* = 43 in 2024, and *n* = 31 to *n* = 35 in 2025. Response counts do not include respondents who submitted the survey but did not answer the question. Figure 1 long description.

**Figure 2.**
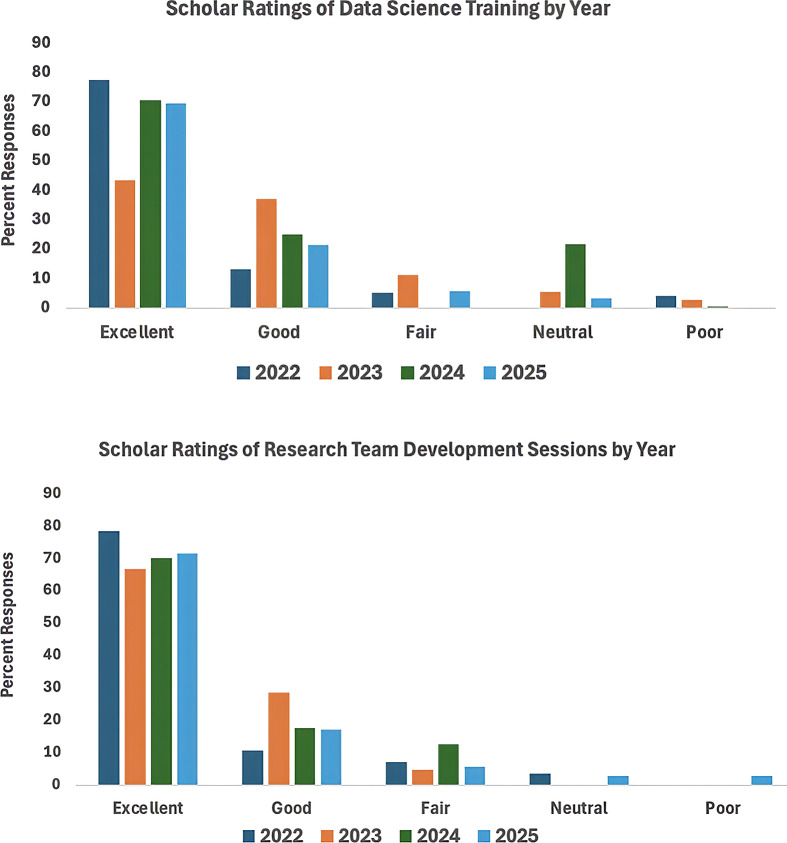
Annual evaluation results from *the All of Us Evenings with Genetics Biomedical Researchers*, Faculty Sumit Data Science Training and Research Team Development Sessions. For the data science training, there were three sessions in 2022, four in 2023, four in 2024, and four in 2025. Response counts across data science training sessions varied from *n* = 25 to *n* = 26 in 2022, *n* = 22 to *n* = 30 in 2023, *n* = 40 to *n* = 42 in 2024, and *n* = 23 to *n* = 35 in 2025. Response counts for the research team development sessions were *n* = 28 in 2022, *n* = 21 in 2023, *n* = 40 in 2024, and *n* = 35 in 2025. Figure 2 long description.

Scholars’ ratings of the plenary sessions are presented in Figure 1. Across all four years, most reported “learning a great deal” for their research and careers, highlighting the program’s relevance and potential long-term impact. The BRFS core components: data science training (A) and research team development (B) were also evaluated (Figure 2). Data science training occurred throughout the BRFS, while team development was primarily completed in one day with ongoing support. Detailed analyses of each BRFS session and those from the One-Day Retreat will be shared in future reports. Overall, scholars consistently rated both components as “excellent” across all cohorts.

### All of Us *BRSP*

To promote the sustainability of research teams formed during the BRFS, the twelve-month *All of Us* BRSP was designed to provide ongoing data science and career development through seminars, as well as continued mentorship and feedback via monthly and ad hoc meetings in which scholars shared progress on team-based projects initiated at the BRFS. Research teams were invited to apply for seed award funding to support the advancement of their projects. Three tiers of seed awards were offered, with maximum funding amounts of $30,000, $15,000, and $2000. In January, the scholars reconvened in Houston for a One-Day Retreat to facilitate another face-to-face meeting for the teams, share research updates, and continue developing their research projects.

Research team outputs included research projects resulting in presentations and publications, development of science education resources, and implementation of community-based public health initiatives and fairs. These projects and associated outcomes have been previously described by Coleman et al. and are detailed in Table S1 of Baker et al. [17,18]. Since the publication of Baker et al. [18], additional outcomes include a presentation by a breast cancer research team at the Annual Biomedical Research Conference for Minoritized Scientists (ABRCMS) in November 2025 and the receipt of a $10,000 grant from the Healthy Americas Foundation by a heart disease research team in November 2025.

Beyond team-based outputs, several scholars independently leveraged the Researcher Workbench outside of their primary project teams by authoring SAS Analytics Guides for *All of Us*, contributing to a publication on the deployment of SAS on the Researcher Workbench [25] and integrating Researcher Workbench-based data analysis into undergraduate coursework. In one instance, a scholar mentored an undergraduate student whose independent research using the Researcher Workbench resulted in a peer-reviewed publication [26].

A central goal of the *All of Us Evenings with Genetics* (EwG) program was to increase the number of UR biomedical science researchers actively using the *All of Us* Researcher Workbench; however, changes in federal funding priorities limit our ability to directly attribute changes in Workbench utilization to this program. Nevertheless, publicly reported metrics provide important contextual insight, showing that the number of registered UR biomedical scientists increased from 572 at the launch of our program to 4782 by 2024. While these increases cannot be causally linked to *All of Us* EwG participation, they are consistent with the program’s broader objective of expanding equitable access to genomic research infrastructure and reducing barriers to engagement for UR researchers.

## Lessons learned

### All of Us *EwG campus seminars*

As we engaged MSIs to become familiar with the *All of Us* database and upcoming research initiatives, through the *All of Us* EwG Campus Seminars, faculty expressed a need for introducing undergraduates to research careers. In response, the *All of Us* EwG team launched virtual Student Townhalls organized by the *All of Us* EwG staff and promoted by the faculty. Undergraduates engaged with current graduate students or alumni in multiple disciplines to discuss research career options and goals.

Student Townhalls were held virtually from 2022 to 2024 at UT-RGV, UTEP, HU and PVAMU. Graduate students and alumni from Arizona State University, BCM, Meharry Medical College, University of Houston, PVAMU, and UT-RGV served as mentors. Sessions began with an introduction to the *All of Us* Research Program, followed by two breakout sessions where mentors shared their academic and career paths. Among several notable positive outcomes from the Student Townhalls, a UT-RGV student connected with Dr. Murray’s research on COVID-19’s impact on UT-RGV student mental health and will contribute to the final publication.

### All of Us *EwG BRFS and* All of Us *BRSP*

**Figure 3.**
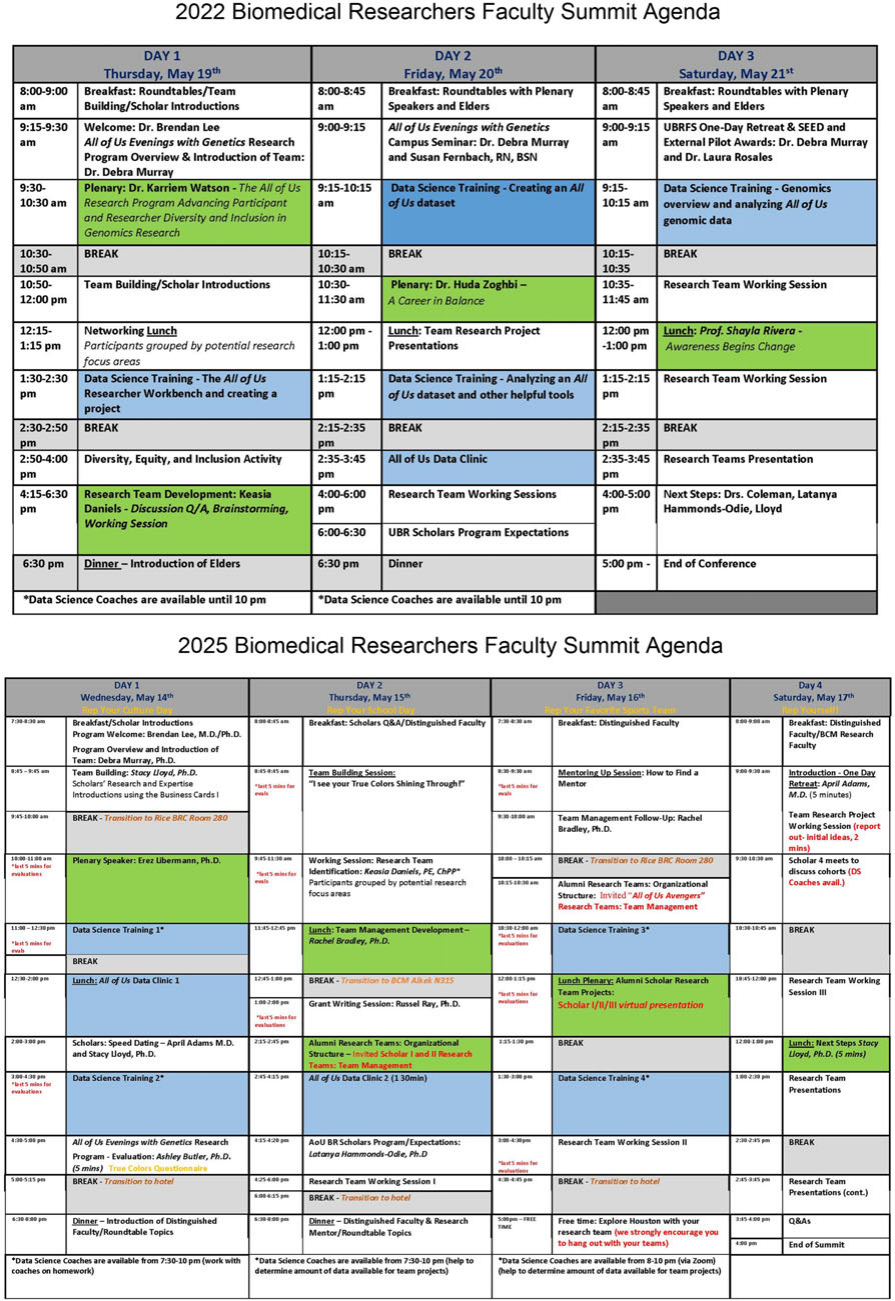
*All of Us Evenings with Genetics* Biomedical Researcher Summit agenda from 2022 and 2025, depicting the evolution of the program. Figure 3 long description.

At the conclusion of the first BRFS, scholars felt that 3 days were insufficient to absorb the content and develop effective teams. Therefore, in years two through four, we extended the BRFS to four days (Figure 3). With this change, we added an additional data science training and built in an “evening off,” encouraging the teams to explore the city of Houston and giving team members more time to get to know each other personally. At the close of year two (2023), we learned that more attention to team management and operation was required to enhance team sustainability. Several teams struggled because of competing interests for their time, which, at times, contributed to BRSP attrition. Therefore, in years three and four, we added a team management and sustainability lecture to the research team development portion of the BRFS and extended this conversation during one of the monthly meetings during the BRSP. During year three, we learned that effective communication and conflict resolution skills were required. During year four, in the research management and sustainability portion of the BRSP, effective communication and conflict resolution were addressed.

## Conclusions

The *All of Us* EwG Research Program observed positive effects of creating diverse research teams and improving research capabilities within MSIs. Program components strengthened institutional research capacity at four MSIs by fostering new collaborations, expanding access to the *All of Us* Researcher Workbench, and positioning early career researchers for long-term engagement through the BRSP.

The success of the *All of Us* EwG Research Program emphasizes a growing need for expanding opportunities and funding to support multidisciplinary research teams. It also delineates the need for providing graduate and postgraduate training in key areas such as team science, project management, effective collaboration, and conflict resolution. Although “team science” gained traction over the past decade, formal training to build, manage, and sustain teams remains limited.

## Supporting information

10.1017/cts.2026.10763.sm001 Lloyd et al. supplementary material Lloyd et al. supplementary material

## Supplementary material

The supplementary material for this article can be found at https://doi.org/10.1017/cts.2026.10763.

## Figure 1. Long description

Two grouped bar charts display scholar evaluations of plenary sessions from 2022 through 2025. The top chart shows responses to the question, “How much did you learn as a result of the plenary sessions?” Most scholars across all years reported learning “a great deal” or “a lot,” with very few selecting “little” or “very little.” The bottom chart shows responses to the question, “How useful were the plenary sessions to your research or career?” Most scholars rated the sessions as “extremely useful” or “somewhat useful,” with very few indicating the sessions were “a little useful” or “not useful.” Overall, the figure demonstrates consistently positive scholar evaluations of the plenary sessions across all four program years.

Navigate back to Figure 1..

## Figure 2. Long description

Two grouped bar charts summarize scholar evaluations of data science training sessions and research team development sessions from 2022 through 2025. The top chart presents ratings for data science training sessions, with most scholars rating the sessions as “excellent” or “good” across all years, and relatively few responses categorized as “fair,” “neutral,” or “poor.” The bottom chart presents ratings for research team development sessions, which were also predominantly rated as “excellent” or “good” across all cohorts. Overall, the figure demonstrates strong scholar satisfaction with both data science training and collaborative team development components of the program.

Navigate back to Figure 2..

## Figure 3. Long description

The figure displays side-by-side visual schedules of the 2022 and 2025 Biomedical Researchers Faculty Summit agendas, illustrating the evolution of the program over time. The 2022 agenda spans three days and includes scholar introductions, scientific plenary sessions, data science training, team-building activities, and research presentations. The 2025 agenda expands to four days and includes additional programming focused on team management, communication, conflict resolution, research sustainability, grant writing, mentoring, and expanded data science instruction. The comparison highlights the program’s growth in duration, structure, and emphasis on long-term team development and scholar support.

Navigate back to Figure 3..
