# Supplementary material for: The All of Us Evenings with Genetics Research Program
Source: J Clin Transl Sci. 2026 Jun 16;10(1):e125. doi: 10.1017/cts.2026.10763 (PMC13420164; doi:10.1017/cts.2026.10763)

**The *All of Us Evenings with Genetics* Research Program**

**SUPPLEMENTAL DOCUMENTATION**

Stacy Lloyd, PhD^1^, Jasmine Baker, PhD^2^, Carolina J. Jorgez, PhD^3^, Julie Coleman, PhD^4^, LaTerrica Williams, PhD^2^, April Adams, MD^5^, Ashley Butler, PhD^2^, Elizabeth Atkinson, PhD^4^, Shamika Ketkar, PhD^4^, Steven Scherer^4^, PhD, Kim C. Worley, PhD^4^, Susan Fernbach, RN^4^, Keasia Daniels, PE, ChPP^6^, Latanya Hammonds-Odie PhD^7^, Laura Rosales, ED^4^, Brendan Lee, MD PhD^4^, and Debra Murray, PhD^4^

**Supplement Figure 1: NIH Racial/Ethnic and Disadvantage Definitions (active links no longer available).**


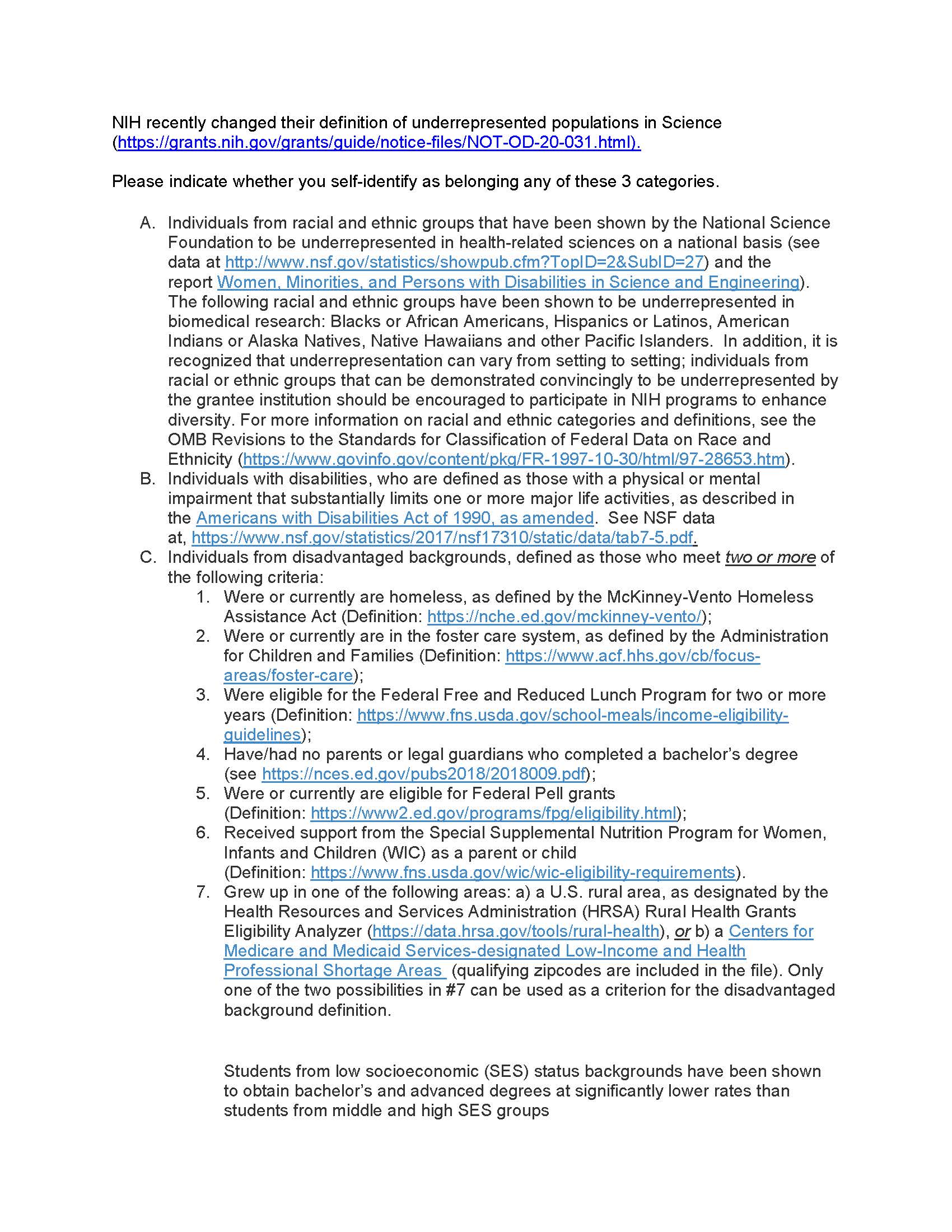


**Supplemental Figure 2. Distribution of Scholar race and ethnicity across cohort years**

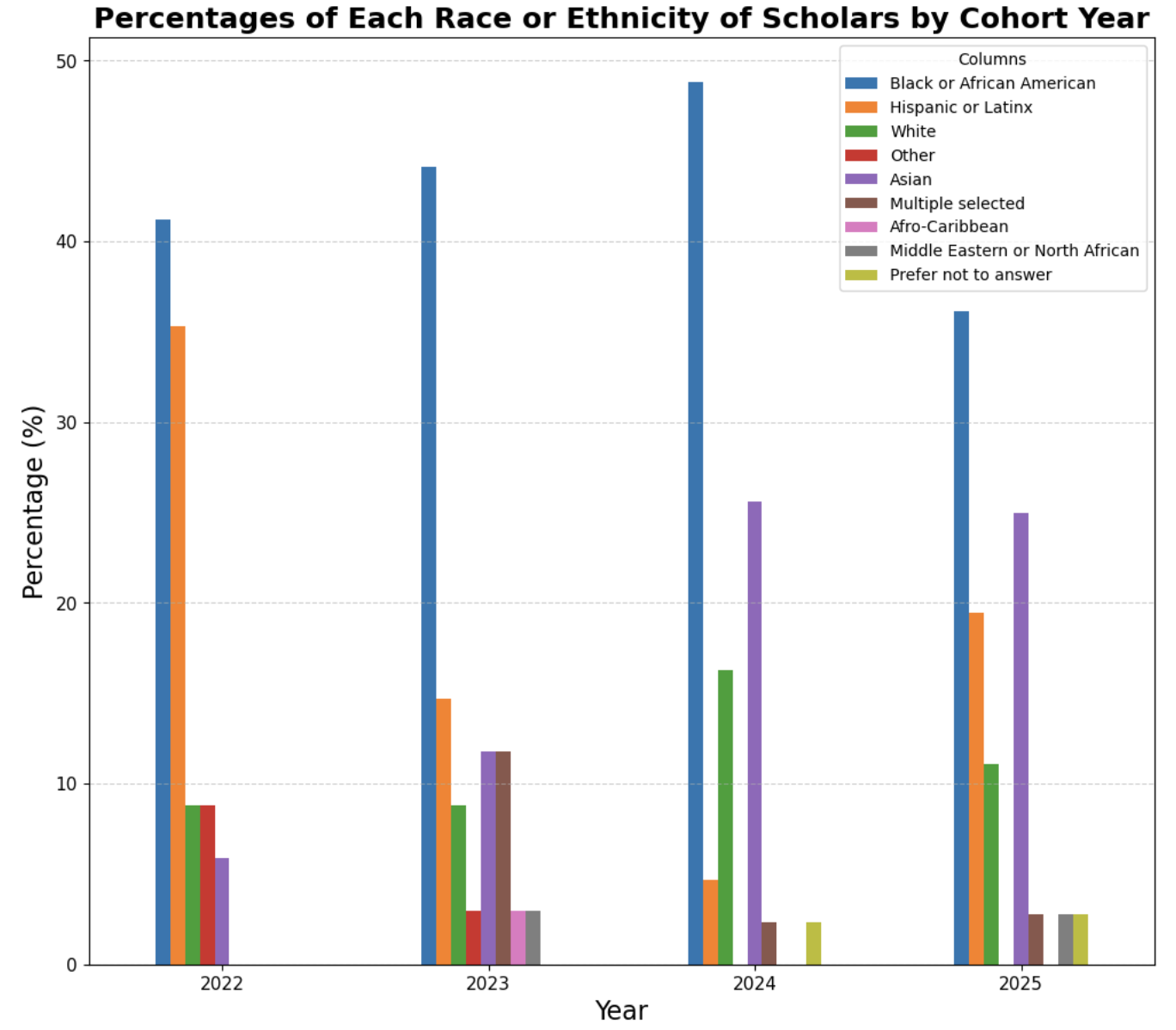


**Supplemental Figure 3. Distribution of *All of Us* Biomedical Researcher Scholar Institutions by Carnegie Classification**


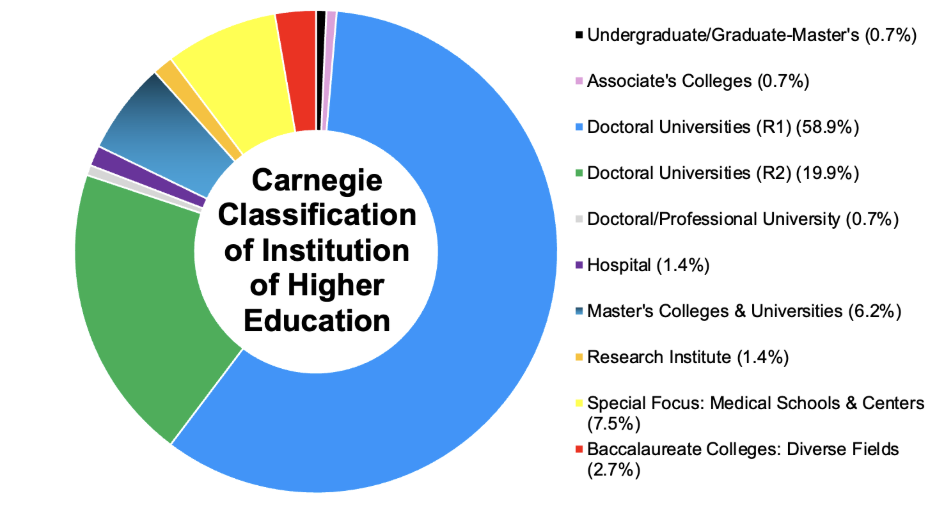

Supplement: Lloyd et al. supplementary material [file S2059866126107638sup001.docx]
